# Male infertility a need for facilitative and supportive nursing care: a literature review

**DOI:** 10.1186/s40834-026-00432-4

**Published:** 2026-02-17

**Authors:** Kevin Gormley, Jameela Saeed Aldossary, Jolly Peter Isaac, Malaika Susan Peter

**Affiliations:** 1 School of Nursing and Midwifery, Queen’s University Belfast, Belfast, UK; 2 College of Nursing and Midwifery, Mohammed Bin Rashid University of Medicine & Health Sciences, Dubai, United Arab Emirates; 3 School of Pharmacy and Biomedical Sciences, Royal College of Surgeons in Ireland, Dublin, Republic of Ireland

**Keywords:** Male infertility, Psycho- social support, Nurse education and training

## Abstract

**Background:**

There is good evidence of significant advancement in care options for men and couples dealing with infertility. Despite this, services for infertility are often applied to both men and women without regard to sex and gender differences in psychosocial needs and perceptions. As a result fertility support services may not adequately meet the needs of men, in the case of male factor infertility. Research investigations are required to identify gaps in care and opportunities for improvement as well as assess intervention efficacy in methods to improve male specific fertility care. Findings could also support and guide education to equip nurses with requisite knowledge and skills to provide evidence based care to meet the needs of this specific patient group. The purpose of this literature review was to explore the opportunities for specialist nurses to offer psychological and social support for men as they deal with infertility.

**Methods:**

A PRISMA systematic approach was selected to undertake a literature review of psycho-social support for infertile men and specialist nursing services to explore potential for improved care. A systematic search was conducted in July 2024, using CINAHL, PsycINFO and MEDLINE electronic databases to identify relevant studies published between the years 2020 and 2024. Using different keywords and mesh terms maximised the search return (including: male fertility, infertility, male reproduction, psychological, social care, fertility nursing). Included studies were required to be in English and address the psycho-social needs and care requirements of infertile men; case studies and literature reviews were excluded.

**Results:**

An initial search offered 195 records: 38 remained after eliminating duplicates and a consideration of title, abstracts and reference lists. 30 full text articles were deemed ineligible (not related to the subject under investigation), leaving 8 included studies. Following a thematic review of these studies (*n* = 8) two themes emerged, that were considered individually and together: (i) social and psychological needs of infertile men; and (ii) specialist nursing services.

**Conclusions:**

The findings from the studies (*N* = 8) demonstrate that men feel excluded from fertility services that are provided for couples. In particular the psycho-social needs of infertile men require specialist assessment, and interventions including counselling and guidance. Nurses working in fertility clinics with specialist preparation and education should involve men throughout fertility treatments, alongside their partners. In summary infertile men value the opportunity for counselling and psycho-social support services that should promote personal confidence to discuss their infertility with partners and families, without either embarrassment or guilt.

## Introduction

Human Infertility is defined by the World Health Organization [1] as a failure of the male or female reproductive system to achieve a pregnancy after 12 months of regular and unprotected sexual intercourse. Estimates suggest that around 10% of couples have fertility issues, and around half are attributed at least in part to the man [2–4]. Male fertility is complex, and contributing causes are generally multifactorial, and an accumulation of inter-related factors including pathological change, social circumstance, personal development, learning and knowledge deficits [2, 5, 6]. The assessment and diagnosis of male factor infertility remain an under researched area of reproductive health [7–9]. The reasons possibly center around perceptions that infertility is a woman’s issue, even where clinical evidence demonstrates a male contributory cause [6–8]. It is argued [10, 11] that despite the prevalence of male infertility it remains relatively invisible and not openly discussed in relation to female infertility (support groups, blogs, and news stories).

Social and psychological circumstance permeates a number of studies that point to a lack of research about male infertility experiences [10, 12]. In a qualitative study exploring men’s’ experiences of diagnosis and coping with male factor infertility [13], participants reported feelings of isolation and failure as a partner. Further studies point out that men tend to adopt a stoic view towards emotional distress, such as male infertility [12, 14, 15]. There is evidence that nurses working in fertility clinics, with training and governance support are well placed to address this service need [12, 15]. This review will explore psychological, and social aspects of male infertility, and consider the potential for specialist prepared nurses to meet this unaddressed and unresolved need in fertility services.

### Study design

A systematic literature review should examine the totality of selected and focused published arguments. A narrative synthesis design was adopted, offering a robust mechanism where findings were discretely synthesised and brought together into themes of academic interest for detailed examination and analysis [16, 17].

### Search strategy

A systematic search was conducted in July 2024, using CINAHL, PsycArticles and MEDLINE electronic databases to identify studies published in peer reviewed journals between the years 2020 and 2024. Included studies required full text availability, publication in English and address the psycho-social needs of infertile men and/or their nursing requirements. Research designs were not limited, reference lists were also considered for possible studies but case studies and literature reviews were excluded (Table 1).

**Table 1:** Exclusion criteria

Study Selection Guide	Inclusion	Exclusion
Study published in English	✓	
Year of publication Limited published date	✓	
Peer reviewed academic studies	✓	
Quantitative, Qualitative and Mixed method design	✓	
Case studies literature reviews		✓

### Study selection

**Fig. 1:**
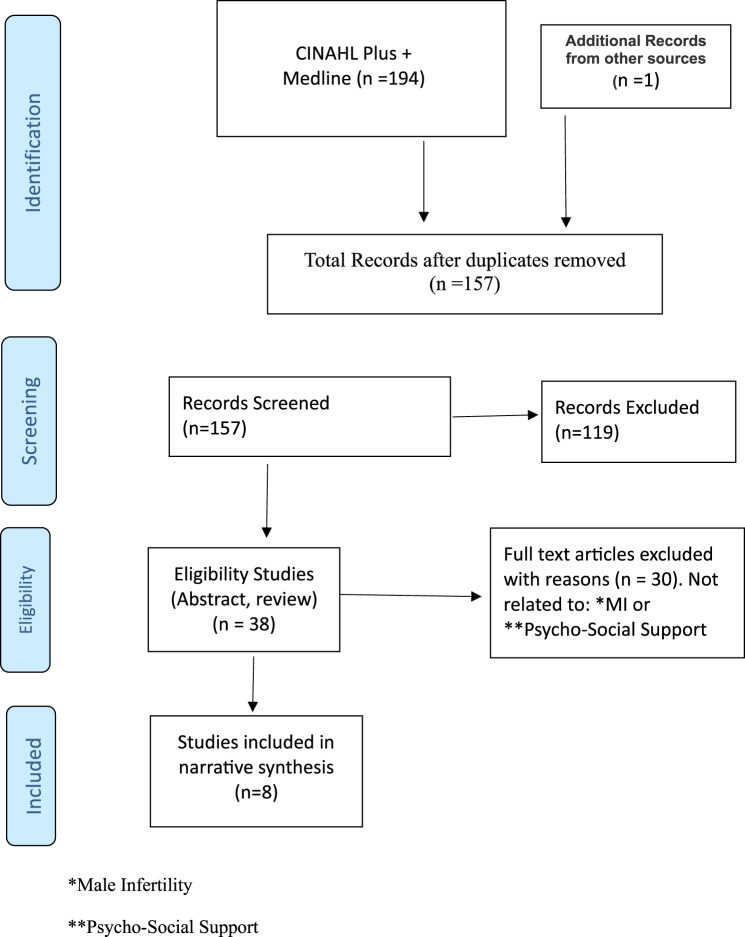
PRISMA flow diagram. *male infertility **psycho-social support

The authors adopted PRISMA (Preferred Reporting Items for Systematic Reviews and Meta-Analyses) guidelines to review selected studies that addressed psycho-social support for infertile men and nursing services to explore service improvement opportunities [16–19]. Using PRISMA facilitated refinement (reducing the number) and alignment (matching objectives and content) of studies with the review (Fig. 1). Flexibility was adopted by the authors in the use of acronyms, and various keywords/mesh terms to maximise the return of studies (including: male fertility, infertility, male reproduction, psychological, social care, fertility nursing). Several excluded papers were deemed ineligible (not meeting the inclusion criteria or examining specifically psycho-social needs of infertile men and/or supportive nursing care). 195 records were identified through database searching and after eliminating duplicates, the abstracts and titles of the remaining 157studies were screened from which 38 studies were shortlisted. A full-text eligibility analysis was then conducted, from which 30 were eliminated, leaving a final eight studies (Fig. 1).

### Quality appraisal

Two authors independently assessed and rated each of the 8 selected studies using an amended check list based on a critical appraisal tool [18–20], which was selected because of its transferable use across different research methodologies. The checklist enabled a structured appraisal of the 8 studies using nine separate sections (including methods, data, sampling, implications) that were rated on a scale of 0 (poor) to 3 (very good) meaning a maximum possible score of 27 (Table 2). The author’s agreed ratings for each study ranged from good [23] to very good [24] and as a result all 9 were deemed appropriate for the literature.

**Table 2:** Included articles and quality checklist

Author, Country, Study Design & Citation Number	Aims	Sampling & Data Collection	Findings	Quality Approval total score allocation max score +27 Hawker et al. (2002)
1 Szatmári, et al. [20], Hungary, Correlation study Cite 22.	This study aimed to elaborate on the effects of the treatment and experiences, to process information, to develop adaptive coping strategies against stress and to indirectly or directly change health behaviours influencing reproduction	Patients with male factor infertility were divided into an observed group (*n* = 57) and a control (*n* = 51) researchers tested their reactions to, and awareness of, the condition.	Study found that counselling of clients with infertility problems a more favourable mental well-being can be established by the active participation of professional helpers. Patients might receive effective, targeted and problem-specific help.	25
2 Kazemi et al. [21] Iran, Cross sectional study Cite 17	This study aimed to develop a theoretical framework for the relationships between coping, adjustment toward infertility, depression and anxiety and to present a confirmatory analysis of the developed theoretical framework among men who, together with their spouses, were candidates for assisted reproductive treatment.	This study was conducted using 212 men selected from the couples who were candidate for ART. Depression and anxiety, adjustment toward infertility, and coping strategies were assessed using self-report questionnaire.	The study showed that higher depression and anxiety scores were related to lower adjustment to infertility. Higher adjustment score was associated with lower self-blame, self-focused rumination, active confronting and avoidance coping strategies scores significantly	24
3 Kyoko et al. [22] Japan Quasi experimental study Cite 18	The present study aimed to examine the effects of a spousal support program to enhance the QoL of male patients undergoing infertility treatment.	This study was conducted among 38 infertile couples in Tokyo (Japan)	The spousal support program was well-received and significantly improved part of the QoL of men who were infertile due to various causes.	25
4 Kyoko et al. [23] Japan clinical trial Cite 20	This study explored the effectiveness of a web-based partnership support program in preventing quality of life deterioration and reducing emotional distress in men undergoing infertility treatment.	a non-randomized controlled trial involving 151 infertile couples in Japan from January to April of 2022. The program consisted of couple discussion, information provision for couple cooperation, and communication techniques.	The study found that a web-based partnership support program was effective in preventing the deterioration of the quality of life of only men undergoing non-assisted reproductive technology treatment	25
5 Abdullahzadeh et al. [24] Iran Qualitative study Cite 21	This study investigates the experiences of men dealing with infertility. The study brings attention to the challenge that men with primary infertility encounter and highlights the importance of culturally sensitive care from healthcare professionals, emotional support, counselling services and public awareness to reduce any potential for stigma surrounding male infertility.	A team of nurse researchers conducted this research in Iran to examine the experiences of 11 men with primary infertility. The participants were selected through targeted sampling and underwent in-depth semi-structured interviews.	The study found that it is important to raise public awareness and provide education about male infertility. Nurses must exhibit cultural sensitivity when caring for men experiencing infertility. Policymakers need to implement strategies to reduce the stigma surrounding male infertility.	26
6 Obst et al. [25] Australia Mixed methods Cite 19	This study aimed to identify the associate risk factor in diagnosed infertility among Saudi men and were based on assessment of laboratory reports.	The team conducted a mixed-methods study utilizing a combined sequential, concurrent design (online survey/semi-structured interviews). Survey outcomes (*N* = 12) were analyzed using quantitative data analysis, while qualitative survey data (*N* = 5) was analyzed by reflexive thematic analysis.	This study found that most men reported that their information and support needs were only somewhat, slightly or not at all met. Preferred information sources on male infertility were a dedicated online resource, app, or fertility doctor/specialist, while support was preferred from fertility specialists and partners.	23
7. Brauner et al. [26] Denmark Cross sectional study Cite 10	The study aimed to assess the association between psychological stress and male factor infertility as well as testicular function (semen quality, serum reproductive hormones) and erectile dysfunction	Participants completed a questionnaire on health and lifestyle, including a 14-item questionnaire about self-rated psychological stress symptoms and stressful life event (SLEs), had a physical examination performed, delivered a semen sample and had a blood sample drawn.	Infertile men reported a higher number of SLEs than fertile men but did not report more psychological stress symptoms. Distress and SLEs were not associated with reduced male reproductive function.	25
8 Hanna et al. [27] UK qualitative questionnaire study Cite 30	This article reports findings from a qualitative questionnaire study focusing on a sample of men with a male factor infertility diagnosis; an under-researched and marginalised group in the context of reproductive medicine.	Participants completed a qualitative questionnaire study that focused on men with a male factor infertility diagnosis;	The study found wide societal discourses presenting men as (unproblematically) fertile, uninvested in parenthood and stoic in their approach to emotional distress. Such norms also ensured that reproduction continues to be presented as a ‘women’s issue’ which burdens women and marginalises men. In understanding male factor infertility experiences, the damaging nature of the social construction of male fertility was clearly illuminated.	24

### Data extraction & results

The purpose of a narrative synthesis is to extract the most important detail from each study and provide a unique and collective interpretation of the studies [17, 19, 20]. In advance the authors agreed a flexible outline of possible areas that aligned with the authors preparatory work (reading, discussions, professional knowledge) and the purpose of the review (Table 3). Two authors read and re-read each of the studies (KG, JP) and agreed two overarching themes that were auditable (records), and based on extracted data from all included studies, they were: (i) Psycho-social needs of Infertile Men and (ii) Specialist Nursing needs.

**Table 3:** Emerging areas

Outline of Data Extraction Areas	
Psycho-social needs of Infertile Men	✓
Extended nursing services for Infertile men	✓
Specialist nursing preparation for infertile men	✓
Clinical governance & evaluation	✓

### Theme 1 psycho-social needs

Societal norms seem to accept reproduction including conception as principally a women’s concern, and when things go awry it is perceived as their problem, which serves to marginalize men even further when they should be recognized as a significant contributor (). Infertility seems to generate a perception among infertile men of being deficient in their societal role which frequently impacts their psychological wellness. This review demonstrates that marginalizing infertile socially results in change to their expected roles. For example, infertile men reported the presence of psycho-social issues caused lowered self-esteem, feelings of inadequacy and social failure, particularly in roles linked to “fathers to-be”. While this is not new there does remain a continued negativity towards men finding themselves in these circumstances, much of which relates to a fact that they were not able to talk about their infertility either with other men or their partners [20, 21, 23, 27]. The review indicates that psycho-social care for infertile men is not effective and often avoided. Infertile men report exclusion from therapeutic conversations with clinical staff and point to the presence of stress leading to reduced self-esteem, depression, isolation, grief, and stigma. These symptoms are further exacerbated, during investigations and treatment where men report associated sexual disfunction that further increases stress. Being able to communicate well is of special social importance as it was clear that men who could effectively use coping strategies by seeking others’ support, reconstructing their goals and expressing of emotion were protected from depression and better adapted to the infertility treatment [21, 23, 25, 27]. Counselling and other forms of emotional and social support would provide infertile men with a source of encouragement and help in articulating concern and reciprocally, nurses with advanced preparation and expertise should be able to anticipate offer appropriate and meaningful support on demand.

### Theme 2 specialist nursing

Male infertility clearly impacts mental and social health and wellbeing, and it is evident that all phases of fertility during investigation and treatments nurses and clinicians should support the couple but ensure that both men and woman are individually assessed and evaluated. Nurses working independently and as part of the interdisciplinary team should ensure that men are fully engaged and involved in treatment plans alongside their partners, which should lead to a reduction negative psycho-social outcomes for infertile man [22, 24, 25]. Nurses particularly should be encouraged to promote communication between men and their partners at the very least to avoid blame scenario for a couple’s inability to conceive or have a child. In a Danish study [26] 10 men receiving treatment at a fertility clinic were interviewed. The participants reported two themes i) a desire for care that captures the need for male specific support and ii) specific services for men with a positive ethos. Men when dealing with infertility clearly require detailed information and encouragement around the complete treatment process, and they must be treated as equal partners to their spouse. There is a demand to see fertility care for couples rather than purely the woman and a service that reflects men’s requirement for psychosocial or emotional care to deal with anxiety or confusion to be recognized and addressed [23–25,27]. Men’s psychological needs seem to be unrecognized, and this creates an imperative for nursing and the interdisciplinary team to be considerably more sensitive to psychosocial aspects of care that are specifically linked with infertile men including low confidence and low self-esteem. It is arguable that in offering such new and innovative support, nursing and health care services would at least contribute towards minimizing the duration and impact of unaddressed male infertility, and in due course maximize potentials for successful conception [22, 26].

## Discussion

The significant prevalence of male infertility when mixed with persistent societal misunderstandings and misconceptions around the cause of fertility and treatment options has led to heightened anxieties for men and couples, which is exacerbated through inadequate or absent nursing care planning. This is particularly evident and the situation more complex in circumstances where the cause of infertility is related to the man. In these circumstances where there is a patent need for sensitivity and support for couples, but men in particular nurses need to respond meaningfully and appropriately with positive support and accurate information. Reproduction treatment and services to date have avoided the discrete needs of infertile men in helping them to either accept or deal with infertility. Recognizing the psycho social impact of male infertility requires a health care response particularly among frontline nursing services who are particularly well placed and should be poised to provide specific psycho-social support and help throughout male infertility investigation and treatment. Much is happening internationally to raise the profile of this matter, which is commendable. More does needs to be done particularly at the micro or clinical level to, to realistically raise the expectations of infertile men [1, 2, 5]. Nurses with specialist training and expertise should be prepared with advanced roles and poised through effective governance and system support to take the responsibility in supporting infertile men in these circumstances.

## Conclusion

Specialist nursing services for infertile men needs to be offered within a milieu that is positive, open, welcoming and nurturing continued support for men to encourage understanding and adherence to treatment and the greater potential for a success. It is imperative that nurses with specialist training and responsibilities should adopt strategies purposefully designed to alleviate social psychological and personal pressures on infertile men. Nurses offering these services will require training, education and professional development opportunities to meet these needs. Infertile men would benefit through counselling and support enabling them to be open and discuss with partners and family their concerns about infertility without either embarrassment or unnecessary guilt. This review clearly highlights the need for greater psychological and social care for infertile men, and this should be routinely available and provided by nurses and other health care services in clinical settings for infertile men. From this basis this review provides the following recommendations:

i) Nurses, and health care professionals working in fertility clinics should provide counselling for infertile males to ensure they receive meaningful advice and helpful information about infertility and that that is appropriately presented.
ii) Nurses should commit to a caring philosophy of openness and ensuring that infertile men are kept fully informed with the process and outcomes of diagnostic investigations and treatment options.
iii) Clinically based research investigations are required to provide realistic evaluations of service for infertile men and their families

## Limitations

The authors recognise the limitations to this work, these include: recent studies could be excluded; three databases were selected; and inclusion criteria requires studies to be published in English. In addition the interpretation of findings of studies is subjective and open to variance to the views of the authors.

## Data Availability

No datasets were generated or analysed during the current study.
